# Massive hemothorax in a pregnant patient with neurofibromatosis type 1

**DOI:** 10.1186/s13019-021-01504-z

**Published:** 2021-04-30

**Authors:** Kumiko Hashimoto, Yuji Nomata, Takayuki Fukui, Akira Takada, Kunio Narita

**Affiliations:** 1 Department of Thoracic Surgery, Toyohashi Municipal Hospital, 50 Hachiken-nishi, Aotake-cho, Toyohashi, Aichi 441-8570 Japan; 2 Department of Thoracic Surgery, Nagoya University, Nagoya, Japan; 3 Department of Radiology, Toyohashi Municipal Hospital, Toyohashi, Aichi Japan

**Keywords:** Hemothorax, Pregnancy, Emergency

## Abstract

**Background:**

Reports of spontaneous hemothorax in patients with neurofibromatosis type 1 are scarce despite the severe complication. We herein present the first case of hemothorax in a neurofibromatosis type 1 patient during pregnancy and discuss the difficulty associated with its diagnosis and treatment.

**Case presentation:**

A 39-year-old female at 34 weeks gestation presented with sudden left back pain and dyspnea. Chest radiography revealed massive left pleural effusion. Computed tomography showed bleeding from the intercostal artery. Although the patient appeared hemodynamically stable, the fetus was in a critical condition. Emergency caesarean section was performed within 1 hour. Subsequently, we performed endovascular coil embolization of the intercostal artery. While this intensive treatment saved the patient, her fetus could not be rescued.

**Conclusions:**

Patients with neurofibromatosis type 1 may develop massive hemothorax without gross lesions. In late pregnancy, sufficient infusion and quick hemostasis are essential and can be lifesaving.

## Background

Spontaneous hemothorax is rare, and case reports on this condition are scarce [1]. Physiological changes in the perinatal period are thought to be one of precipitation factors for hemothorax in patients with pulmonary arteriovenous malformation and hereditary telangiectasia [2]. However, there have been only three reports of spontaneous hemothorax in patients with neurofibromatosis type 1 (NF1), all of which occurred in the postpartum period. Herein, we present the first case of hemothorax during pregnancy in a patient with NF1.

## Case report

A 39-year-old female at 34 weeks gestation presented with sudden left back pain and dyspnea. She had been diagnosed with NF1 over 20 years prior. She had no family history of NF1 and no history of chest trauma, prior surgery, or catheter insertion. She was being treated for gestational hypertension.

**Fig. 1:**
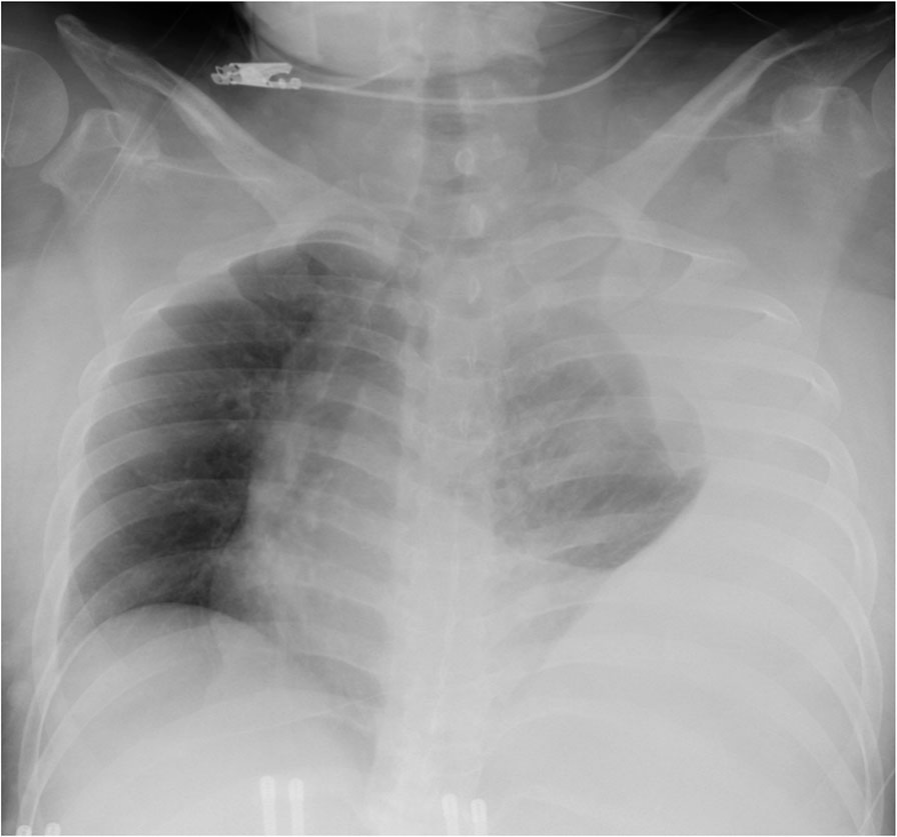
Frontal chest radiograph showing massive left pleural fluid collection with a mediastinal shift to the right

**Fig. 2:**
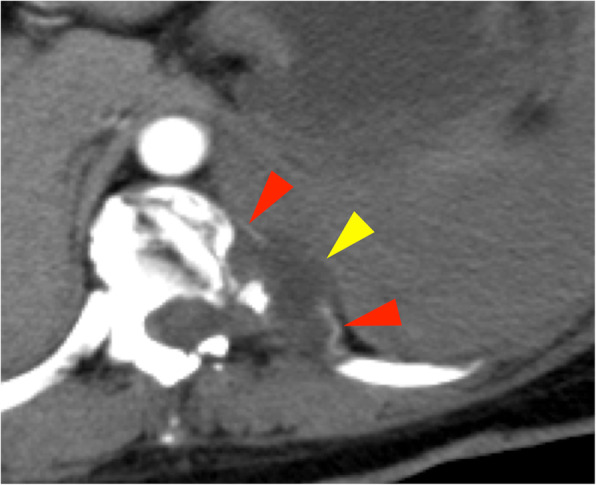
Chest computed tomography showed a massive hemothorax and an irregularly shaped left 10th intercostal artery (red arrow). Intrathoracic dural ectasia was observed close to the artery (yellow arrow)

On arrival, she was conscious with a blood pressure of 110/90 mmHg and pulse rate of 90 beats/min. Her hemoglobin level was 10 g/dL and oxygen saturation was 97% with 10 L/min oxygen delivered via mask. Chest radiography revealed a massive left pleural effusion with left-to-right mediastinal shift (Fig. 1). Chest computed tomography (CT) revealed a left pleural effusion and irregularities of the left 10th intercostal artery (Fig. 2). We suspected hemothorax because the CT value of the effusion was 45 Hounsfield units. Furthermore, CT findings also implied intrathoracic dural ectasia close to the artery. A chest tube was inserted immediately; however, only 400 mL of old blood could be removed, and no further significant discharge was collected though the pleural effusion remained. The patient was hemodynamically stable following fluid resuscitation; however, the fetal heart rate was decreasing, and the fetus was in a critical condition. After obstetric consultation, we performed an immediate caesarean section under general anesthesia; the operative time was 40 min and the volume of blood lost including amniotic fluid was 300 mL.

**Fig. 3:**
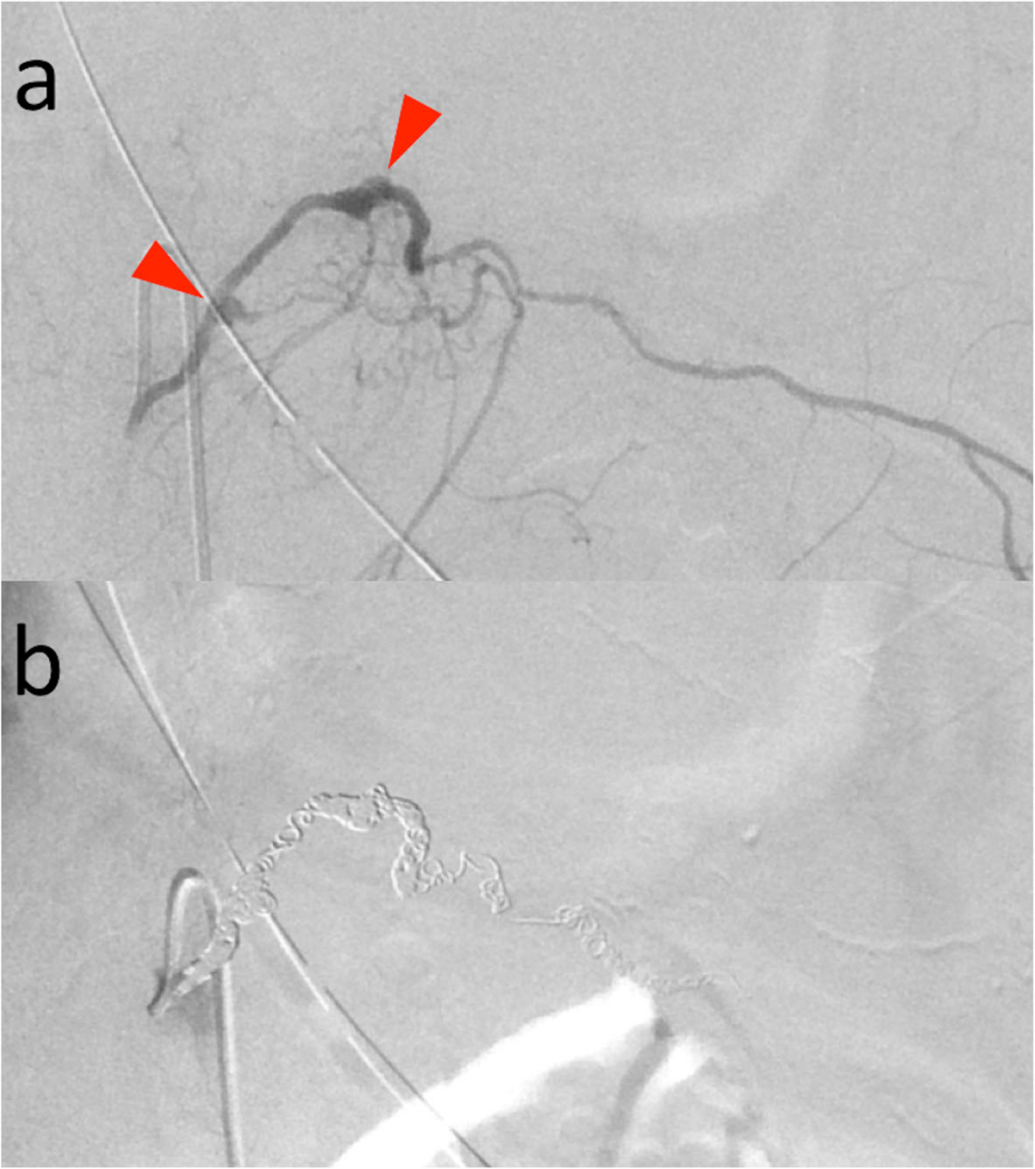
**a** Selective left 10th intercostal arteriography demonstrated irregular vessels and small aneurysms (red arrows). Extravasation of contrast agent was not detected. **b** Left 10th intercostal arteriography after coil embolization showed complete occlusion of the aneurysms

Selective angiography revealed irregular vessels and small aneurysms of the left 10th intercostal artery (Fig. 3a). Extravasation of contrast agent could not be detected. Therefore, we embolized the irregularly shaped portion of the artery. The distal site was embolized using 20 micro coils: 6 Orbit Galaxy (Codman & Shurtleff, Raynham, MA), 6 Tornado Embolization Coils (Cook Medical, Bloomington, IN), 6 Azur Embolization System CX18 (MicroVention Inc., Aliso Viejo, CA), and 2 Interlocking Detachable Coils (Boston Scientific Corporation, Marlborough, MA). The proximal site was occluded using 3 Orbit Galaxy Microcoils. The artery and aneurysms were all embolized successfully (Fig. 3b). The duration from hospitalization to completion of treatment was about 5 h.

The patient’s condition improved postoperatively. Her blood pressure after caesarean section was 90/60 mmHg. After embolization, it increased to 160/80 mmHg. Although the intensive treatment saved her life, the fetus could not be saved. Twenty days after embolization, lest pleural cavity decortication was performed and about 2500 g of hematoma was removed; the constriction of the left lung was released. One month post embolization, the patient was discharged in good condition. No further aneurysms or irregular arteries were found on full-body contrast-enhanced CT before discharge. No symptoms of recurrence ware reported during the next 18 months, and we followed up with CT regularly.

## Discussion and conclusions

The autosomal dominant genetic disease NF1 affects approximately 1 in 3000 births [3]. Patients often suffer from malignant neoplasms and various other comorbidities [4–6], thus requiring continuous medical support. Vasculopathy is less common though severe complication with an incidence of only 3.6% [7]. The occurrence of hemothorax in the perinatal period has been reported in only three cases of NF1, all postpartum [5, 6]. To the best of our knowledge, this is the first case of massive hemothorax in a pregnant patient with NF1.

In small arteries such as the intercostal artery, invasion by neurofibroma causes intimal thinning of the media, elastic fragmentation, and aneurysmal dilatation, which are influenced by the fragile nature of the vascular tissue [8]. Vascular lesions of small arteries are often asymptomatic and may thus go undetected before severe complications occur.

Hemothorax develops when other factors, such as tumors and aneurysms, overlap with vascular vulnerability [4]. Furthermore, pregnancy might be considered a precipitating factor for hemothorax in patients with NF1. Pregnancy exacerbates NF1 itself [5, 6]; furthermore, increased intrathoracic pressure, blood volume, and cardiac output lead to increased blood pressure. Elevated steroid hormone levels also increase vascular fragility.

Hemorrhagic shock may be overlooked in late pregnancy. Blood volume in late pregnancy is 40–50% higher and the blood pressure may drop after 40% of circulating blood volume (1.5 L) is lost [9]. The fetus is directly affected by this decreased blood flow. In our case, the patient seemed hemodynamically stable despite persistent bleeding. Therefore, we focused on the fetus and performed emergency caesarean section within 1 hour from admission, which was a very high risk procedure for the patient.

Rapid infusion in the emergency room usually targets a blood pressure of 100 mmHg. However, this infusion was insufficient in our patient to maintain fetal circulation. More infusion may have improved the fetal condition and caesarean section could have been avoided. Embolization should be performed immediately with adequate infusion. In our patient, the time to embolization was only 90 min. Her blood pressure improved to 160 mmHg after embolization, indicating the severity of bleeding. Complete bleeding could be halted after 5 h from the time of arrival and there is a dire need to significantly cut down this timing to achieve better outcomes.

For hemorrhagic shock associated with obstetrical bleeding, transport to the operating room and emergency caesarean section should be a priority. However, since our patient presented with hemorrhagic shock from an unknown cause, scrutiny and discussion with obstetrician were necessary. We believe that the fetus could have been rescued if the patient was operated soon after presenting to the hospital. However, embolization delayed more without scrutiny in emergency room.

Several treatments have been reported for hemothorax, including conservative treatment with drainage, thoracotomy, and endovascular coil embolization [3]. In this case, the bleeding site was detected on contrast-enhanced CT; therefore, endovascular treatment was feasible. Surgical repair would not have been safe as the field of view would have been obscured by the massive hematoma and dural ectasia. Furthermore, the proximity of the site of bleeding to the dural ectasia implied a risk of perforation during the procedure. In cases there the site of bleeding extends further to the proximal side, intrathoracic surgical repair is no feasible. Therefore, considering the fragility of the artery, the site of bleeding, and the presence of dural ectasia, surgical repair would have been extremely risky.

This case study demonstrates that, even in cases without gross lesions such as tumors and aneurysms, patients with NF1 have the potential to develop massive hemothorax during the perinatal period. Since it is difficult to prevent this outcome, strict hospitalization or blood pressure control, especially late in pregnancy and postpartum, should be considered in these patients. When massive hemothorax does occur, immediate endovascular treatment with adequate infusion should be preferred.

## Data Availability

The data that support the findings of this report are available from the Toyohashi Municipal Hospital. The author can make it available upon reasonable request.

## Abbreviations

CT: Computed tomography
NF1: Neurofibromatosis type 1
